# Investigating Connections between Need for Cognitive Closure and Climate Change Concern in College Students

**DOI:** 10.3390/ijerph17155619

**Published:** 2020-08-04

**Authors:** Margaret Orr, Alan Stewart, Andrew Grundstein

**Affiliations:** 1 Department of Communication, George Mason University, Fairfax, VA 22030, USA; 2 Department of Counseling and Human Services, University of Georgia, Athens, GA 30602, USA; aeswx@uga.edu; 3 Department of Geography, University of Georgia, Athens, GA 30602, USA; andrewg@uga.edu

**Keywords:** climate change, psychology, communication, need for cognitive closure

## Abstract

Understanding how people’s worldviews and individual personality differences affect their thinking about anthropogenic climate change is critical to communication efforts regarding this issue. This study surveyed University of Georgia students to investigate the role that need for cognitive closure plays in level of climate change worry. The relationship between these two was found to involve suppression—a subset of mediation—by the social dimension of political conservatism. Political conservatism was also found to play a mediating role in the relationship between need for cognitive closure and support for governmental and personal climate solutions. However, social conservatism played this mediator role in women, and functioned as a suppressor for men. These findings help inform audience segmentation and creation of climate-related messages based on audience worldview and personality.

## 1. Introduction

Despite the vast amount of scientific evidence showing that human activities are causing the Earth’s climate to rapidly change; and that these changes will result in dire consequences in the future, there is substantial doubt and inaction regarding this issue in the United States [1,2] Only about 57% of American adults know that climate change is caused mostly by human activities, and only 49% know that most scientists agree that this is the case [2]. Enough doubt has been created about these facts, some of it purposefully, that the United States, one of the top emitters of fossil fuels worldwide, is making little to no progress to solve this issue [3]. It is of utmost importance for the future of humanity that Americans change their behaviors regarding climate change, from grassroots individual actions to sweeping policy and energy generation changes. A key part of making this behavior change happen is communicating the risks and ramifications of climate change in such a way that people are motivated to make a change. Thus, it is critical to study how climate change is communicated, what messages work for different audiences, and what makes different audiences more or less receptive to climate-related information. One way that audiences can be differentiated is by gender. It has been shown that women generally exhibit greater climate change knowledge and concern than men [4]. However, they also tend to underestimate their level of knowledge more than men do [4]. Another divider when it comes to climate change belief is political affiliation. There has been significant political polarization regarding the climate change issue since approximately 2000 [5]. Much of this partisan divide is based upon fear of increased regulation in conservatives [5]. This polarization makes messaging difficult, as people immediately side with their political allegiance when they hear the words “climate change”. Therefore, it is important to look at other factors that influence climate change beliefs, and maybe even be underlying in political affiliation. One such avenue is individual personality variables, which can play a role in receptivity to and effects of climate change communication.

Individual differences in people’s worldviews can be a major barrier to climate change related communication. Messages that work for someone with one worldview will likely not work for someone with another. One aspect of a person’s worldview is their level of need for cognitive closure (NCC). NCC is defined as an individual’s need for a definite answer to a question and intolerance of ambiguity [6]. The impact of NCC on people’s behaviors and information processing has been studied through a number of different avenues relating to the climate change issue. These include conspiracy belief [7], precautions against long-term or future risks [8], open-mindedness and competing messages [9], and pro-environmental attitudes and behaviors [10].

In a 2017 study, Marchlewska et al. investigated the connection between NCC and belief in conspiracy-related explanations for events. Results of this study showed that there was a positive link between NCC and belief in conspiracies, and this effect was strongest when the conspiracy theory was salient. Results also showed that people high in NCC latched on to conspiracy-based explanations when the cause of an event was uncertain, but not when the cause of the event was certain [7]. This study sheds light on what motivates people high in NCC to adopt a conspiracy-based belief in the face of uncertainty, which may help climate communicators to understand why some people do not believe the uncertainty-laden projected effects of climate change. One example of a conspiracy belief about climate change is “Climategate”, which occurred in November 2009. The Climate Research Unit at the University of East Anglia was hacked, and over 1000 emails between researchers were published. The released emails used scientific jargon, slang terms, and acronyms that raised alarm within the general public, and some sentences were taken out of their proper context by news outlets [11]. An investigation found no fault in the integrity of the research being done by the scientists whose emails had been hacked and published [12]. However, conspiracy beliefs about climate change persist despite this. University of Georgia professor and Weather Channel podcast host Marshall Shepherd has had plenty of experience with such beliefs. “I get emails weekly from people with their theories and thoughts on climate change or why I’m absolutely wrong on something,” he said. These beliefs can come from individuals themselves, or can stem from coordinated misinformation campaigns: “I think you have this sub-group of people in the American population…that very much propagate these conspiracy theories,” Shepherd says, “but then you do have this deeper, coordinated effort to propagate conspiracy….you see certain organizations and think tanks talking about different theories of climate change and that we need to teach alternative perspectives about climate change in schools—that’s coordinated effort, and so we need to understand that” [13]. In their 2010 book Merchants of Doubt, Naomi Oreskes and Erik Conway confirm the presence of intentional climate-misinformation campaigns [3]. Marchlewska et al.’s 2017 [7] study could help us to understand the role that NCC plays in misinformed, conspiracy-based climate change beliefs.

Individual differences in personality can affect whether people tend to take precautions regarding their future. One such precaution is screening for chronic medical problems such as cancer, diabetes, or heart disease. Eiser and Cole [8] investigated how individual differences in young women ages 20–25 affected the behavior of getting a regular cervical cancer screening. Results showed that respondents lower in NCC were less likely to have had a test for cervical cancer or to be planning to get one. The authors say this might mean that for people high in NCC, the resolution of uncertainty in and of itself may be a motive for getting tested. That is, they do not solely seek a negative/all clear result, they want to know if they have something wrong with them. Based on these results, climate change may be able to be framed similarly to a long-term illness like cancer that can be prevented or dealt with efficiently through precautionary action. However, climate change messages exist in a highly competitive environment. While there are messages about how climate change must be dealt with, people are also being bombarded with messages saying that climate change is a hoax. This can cause a lot of conflict for people forming opinions about climate change and climate action strategies.

One aspect of NCC is a person’s open- or closed-mindedness. Nisbet et al. [9] investigated in 2013 how open/closed-mindedness affects attitudes towards climate change in noncompetitive vs. competitive message environments. Low NCC is associated with open-mindedness, and high NCC is associated with closed-mindedness. This study found that when a video message focusing on the positive outcomes of climate policy/action was viewed in conjunction with a video message focusing on negative economic costs of climate policy/action, people who were less open-minded perceived fewer benefits to climate policy and were less supportive of it. On the contrary, those who were more open-minded and viewed the two messages in competition exhibited increased benefit perception and were more supportive of climate policy/action [9]. Results of this study help to reveal how people high in NCC process the often-conflicting messages about climate change that are portrayed by the media, which helps communicators in the effort to garner support for actions against climate change.

Support of climate policy and action, as well as individual behaviors that protect the environment, are critical for the fight against climate change. Panno et al. [10] investigated how NCC affects pro-environmental behavior. This study was done with two surveys. In the first, respondents’ NCC levels and pro-environmental preferences and behaviors were measured, and political orientation was self-reported. This sub-study sought to understand the connection between NCC and pro-environmental behavior, and found that the two were related through political belief. In the second study, participants answered questions to measure their levels of NCC, pro-environmental attitude and behavior, and economic and social political beliefs. This second study found that this relationship was mediated by the social dimension of political ideology—conservatism specifically [10]. Communicators can use these results to target social aspects of conservative political belief in message building, as opposed to trying to cater to the economic aspect as well.

Although NCC has been applied to study pro-environmental behaviors and attitudes, the present study is the first to use NCC to study concern for climate change specifically. NCC was chosen for this study because its use regarding environmental behaviors provides a framework that this study can be modeled after. NCC has also been shown to relate to political beliefs, and climate change is a very politically charged topic in today’s society. Finally, NCC was chosen because climate change can be considered a long-term risk, similar to the risk of cervical cancer—the perception of which Eiser and Cole studied with relation to NCC as described above [8]. Environmental behaviors, political views, and climate/long-term risk perception are all related to the hypothesis described below.

One of the keys to combatting anthropogenic climate change is changing the behavior of the world’s population. Behavior changes can be brought about in a number of different ways. One method of changing behavior is to tailor a message encouraging behavior change towards personality aspects shared by a relatively large group of people. One such personality trait is level of need for cognitive closure (NCC), an indicator of how well a person deals with uncertainty in their life and how quickly they look for a definite answer to an open question. As climate change entails a certain level of uncertainty, and NCC has been studied already with respect to environmental beliefs, NCC presents an interesting avenue of study in the realm of how personality relates to climate change perspectives.

The goal of this study is to examine the relationship between NCC and views regarding climate change; and the relationship between NCC and climate solution support, as well as the role that political beliefs may play in these relationships. There are many ways to segment and investigate audiences when it comes to climate change communication. This study focuses on people who may believe climate fallacies due to their aversion to the uncertainties associated with projected effects of climate change. These uncertainties have been used by climate denial campaigns to discredit scientists; and uncertainty can lead to a lack of self-efficacy in audiences. Therefore, this study focuses on individuals with a high level of need for cognitive closure, and the effects of high NCC on their climate change worry and solution support.

### Research Questions

This study sought to answer the question of whether level of need for cognitive closure (NCC) plays a role in climate change worry (CCW), and whether this relationship is mediated by political views. Additionally, we wanted to explore potential relationships between each of these variables and level of support for various climate solutions.

**Research Question 1**. *How do need for cognitive closure and conservatism affect climate change worry?*
**Research Question 2**: *How do need for cognitive closure and conservatism affect support for climate solutions?*

## 2. Materials and Methods

### 2.1. Survey Methods

In order to investigate these questions, a survey was distributed online via the Qualtrics platform to undergraduate and graduate students at the University of Georgia. As an incentive for participation, students were offered the chance to enter for a drawing of one of five $40 Amazon gift cards. The survey contained four scales, measuring participants’ levels of need for cognitive closure, levels of worry about climate change, levels of support for various climate change solutions, and both the social and economic aspects of their political beliefs. Demographic questions, including gender identification, race, religion, and age were asked at the end of the survey. The University of Georgia (UGA) is a public university in Athens, Georgia, with approximately 30,000 enrolled undergraduate students. Students are mostly white, with Asians, African-Americans, and Hispanics making up most of the rest of the student body. Table A5 and Table A6 in the appendix provide information on the ethnic and religious diversity of our sample. There are approximately 5000 more female-identifying undergraduates than male-identifying. The University of Georgia is involved in the Georgia Initiative for Climate and Society, has a Climate Action Planning Task Force, and runs an Office of Sustainability. Although not all UGA students will encounter climate-change-related information and action during their studies, the university displays a commitment to climate action (ethical approval number: PROJECT00000650, date 18 June 2019).

The 15-item NCC Scale with Weights, created by Roets and Van Hiel [14], was used to measure respondents’ levels of need for cognitive closure. This scale was chosen because it assesses all dimensions of a person’s NCC level while reducing the number of questions from the 41 in their modification of the original scale to a more manageable—15 [6,15]. This allows for multiple scales to be used in this survey without requiring respondents to answer a cumbersome number of questions. Questions in the 15-item NCC Scale with Weights are rated on a 6-point Likert scale from 1 = Strongly Disagree, to 6 = Strongly Agree. Their responses are summed, and higher sums indicate higher NCC. The test-retest stability of this scale is 0.79, and the Cronbach’s alpha is 0.89, which is comparable to the original 41-item scale’s 0.9. Additionally, there is no difference in scale means between the 41-item and 15-item scales [15].

The Climate Change Worry Scale, developed by Stewart [16] was used to measure respondents’ level of concern regarding climate change and its effects. This is a 10-item scale that assesses how often respondents have worries regarding climate change and its effects. Participants indicate how frequently each item, in the form of a statement, applies to them on a five-point Likert scale ranging from 1 = Never to 5 = Always. The Cronbach’s alpha for this scale is 0.95.

Climate change solution support was measured using questions from the Yale Program on Climate Change Communication’s surveys on which their Climate Opinion Maps are based [2]. Eight questions were chosen from the original list of sixteen. This subset was chosen to focus on specific items related to the scale of various climate mitigation actions (i.e., federal, local, and personal), and to limit the number of questions in the survey so that participant fatigue could be minimized. The Cronbach’s alpha of this scale is 0.79.

Respondents self-reported their social and economic political conservatism, respectively, on a scale ranging from 1 = Very Liberal, to 7 = Very Conservative. Self-reporting was chosen to minimize the number of questions in the survey, and to make the distinction between social and economic conservatism easy to find within the data. These questions had been previously used by Cacciatorie, Scheufele, and Corley in their 2011 study [17].

### 2.2. Analysis Methods

The first step in analyzing survey results was to look at first-order correlations between each of the variables studied. These relationships were considered significant at α = 0.05. Following the analysis procedure of Panno et al. [10], these indicators of simple relationships were then used to inform the creation of mediation models. Detailed information can be found in the Supplementary Materials.

A number of different mediation models were created for the first part of this study. Mediation models were used following the example of Panno et al. [10]. While Panno et al. went to a regular regression model before mediation, we were able to move straight to mediation models based on their findings [10]. The first, and most broad model involved CCW and NCC mediated by overall political conservatism for both men and women. This model was subsequently broken down, due to differences between the genders previously noted in the literature [10]. The second set of mediation models depicted the relationship between NCC and CCW as mediated by overall conservatism for men and women, respectively. Finally, a third set of models was created based on Panno et al.’s finding that the social dimension of conservatism played a larger role in the NCC/pro environmental attitude relationship. This third set contained models of the NCC/CCW relationship as mediated by social conservatism for both men and women separately, and economic conservatism for both men and women separately. In total, seven mediation models were a part of this analysis. Coefficients in these models were considered significant at α = 0.05. These models were created in R (version 2.3, R Fundation for Statistical Computing: Vienna, Austria,2006), an open-source statistical software, using the psych, expss, diagram, and lavaan packages.

The second part of this study investigated relationships between level of need for cognitive closure, dimensions of conservatism, and climate change worry with level of support for personal versus governmental climate change solutions. First order correlations between each of these variables were calculated, and similarly to the first part of this study, were used to inform the creation of mediation models. These models were created for the relationship between NCC and personal/government solution support, mediated by overall, social, and economic conservatism, separated between men and women. A total of twelve mediation models were built to analyze each of these relationships. We chose these models because level of support for climate solutions is more behavioral than climate change worry—this allowed us to bring a behavioral aspect to the study, in a similar manner to Panno et al. [10]. R (version 2.3) was again used to create these models, using the psych, expss, lavaan, car, and diagram packages.

In both of these studies, a Sobel test was used to determine which mediation effects were significant. The Sobel test is a special form of a t-test that is used to determine whether there is a statistically significant difference between the direct effect of an independent variable on the dependent variable, and the effect when the mediator is taken into account. This test was chosen because of its wide usage and acceptance in mediation research. The Sobel Test was run using a website widget, which can be found at http://quantpsy.org/sobel/sobel.htm.

## 3. Results

**Figure 1:**
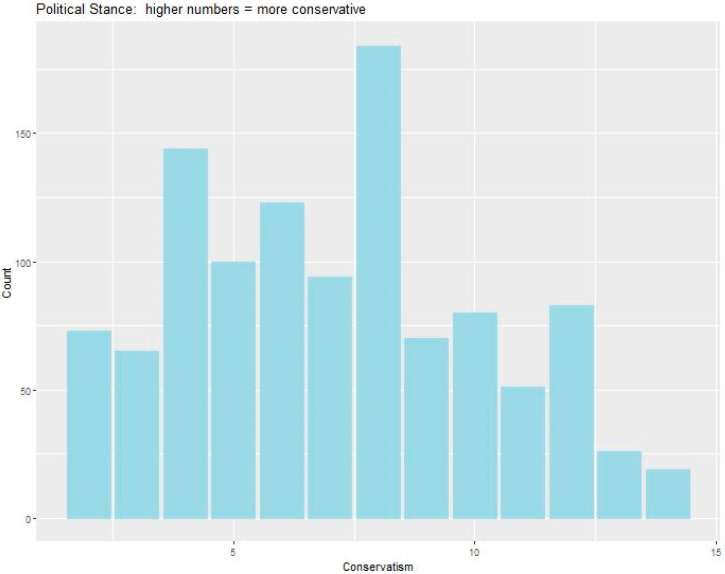
Histogram of political stances. Higher numbers indicate more conservative stances.

**Figure 2:**
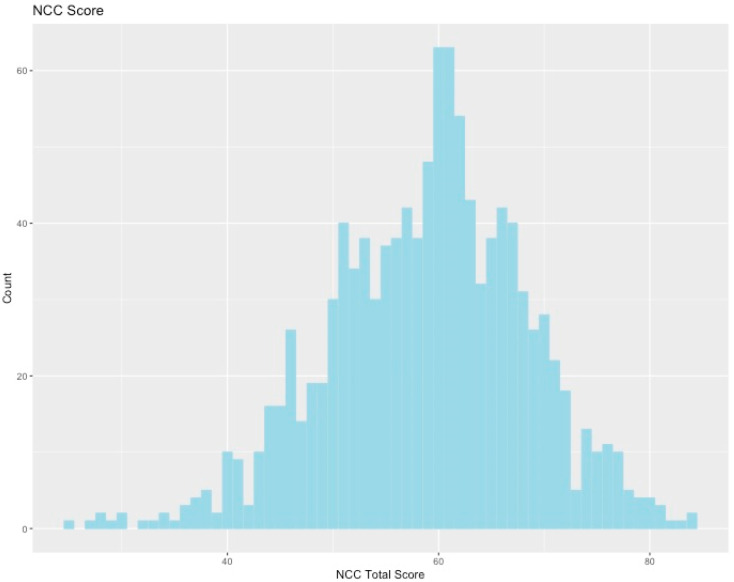
Histogram of scores on the need for cognitive closure (NCC) scale. Higher numbers indicate higher level of NCC.

**Figure 3:**
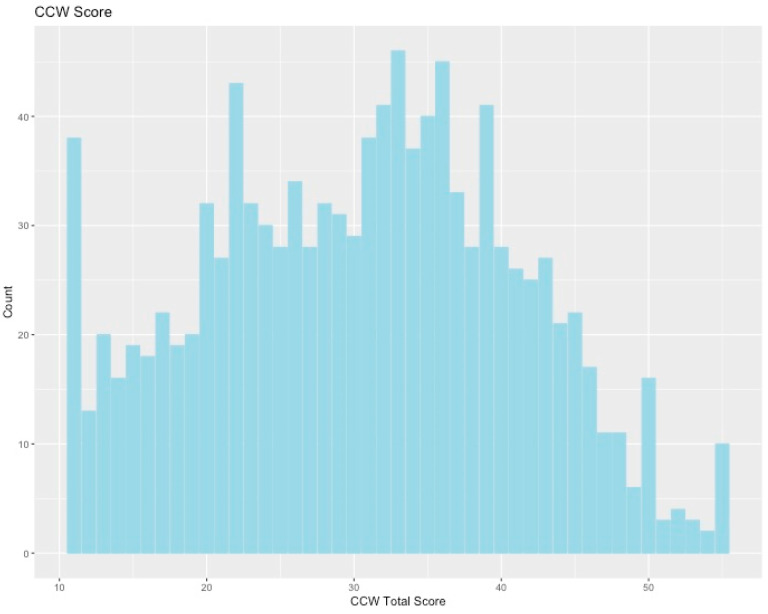
Histogram of scores on the climate change worry (CCW) scale. Higher numbers indicate higher levels of climate change worry.

We received approximately 1100 responses to the survey. Of these participants, 731 were women, 363 were men, and 17 were other/non-binary/preferred not to answer. Respondents were 70% white, 6.5% Latinx, 6.6% black/African-American, and 11.3% Asian/Pacific Islander (5.9% other/prefer not to answer). Most respondents were either in the center of the political spectrum or slightly left of center (more liberal); however, the political spectrum overall was fairly well-represented (51% of the sample on the left, 49% on the right, see Figure 1). Respondents tended towards slightly higher levels of NCC, shown by the slight left skew of the histogram in Figure 2. Level of climate change worry showed a very large spread, with a drop-off at higher scores (Figure 3).

### 3.1. Need for Cognitive Closure, Conservatism, and Climate Change Concern

**Figure 4:**
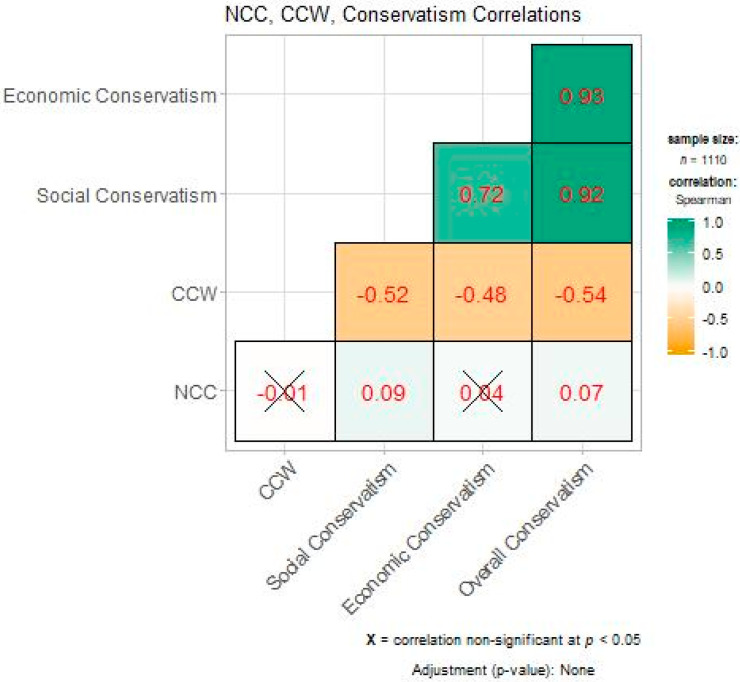
Correlation matrix for climate change worry (CCW), need for cognitive closure (NCC), social conservatism, economic conservatism, overall conservatism. These numbers represent the R^2^ value for each of the relationships.

The matrix of correlations between need for cognitive closure, conservatism, and climate change worry (Figure 4) shows that climate change worry was negatively correlated with all three conservatism items (overall/CCW = −0.54, economic/CCW = −0.48, social/CCW = −0.52). This indicates that climate change worry decreases with increasing levels of all three dimensions of conservatism. The social conservatism correlation is slightly larger than that of economic conservatism. Need for cognitive closure, however, was only significantly correlated with social and overall conservatism, with the slightly larger coefficient between the two also being social conservatism (0.09 vs. 0.07). The correlation between need for cognitive closure and climate change worry (−0.01) was insignificant, as denoted by the “X” in that particular box of Figure 4. Because the direct correlation between NCC and CCW was insignificant, but correlations between NCC/conservatism and conservatism/CCW were, we created mediation models to further investigate how these three variables interact with each other.

**Figure 5:**
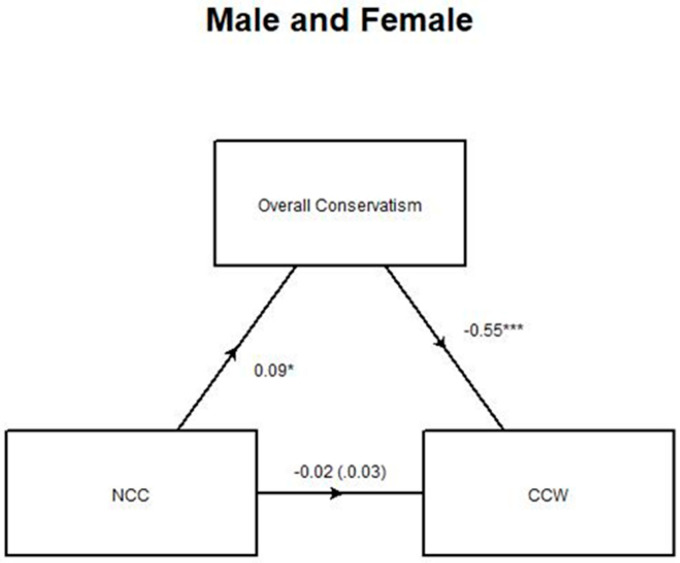
Model of the relationship between NCC and CCW as mediated by conservatism for both men and women. The c’ coefficient is in parentheses. The asterisks denote the level of statistical significance of the observed statistics. The convention is *** *p* < 0.001 and * *p* < 0.10.

A mediation model showing the relationship between need for cognitive closure and climate change worry as mediated by overall conservatism for both men and women (Figure 5) tells a similar story to the correlation matrix (Figure 4). In these models, the *a* coefficient represents the effect of NCC on conservatism, *b* represents the effect of conservatism on CCW, *c* represents the direct effect of NCC on CCW, and *c’* represents the indirect effect of NCC on CCW, which takes the mediating variable into account. There was a strong and highly significant relationship (−0.55, *p* < 0.001) between overall conservatism and climate change worry. The relationship between need for cognitive closure and overall conservatism was smaller in magnitude but still significant (0.09, *p* = 9.786 × 10^−5^). Because of previously described differences between genders [18] this model was broken down into two parts: one using responses from men and the other using responses from women. A small number of responses from individuals identifying as non-binary or “other” (*n* = 17) were received, therefore, these responses were not used in these analyses. Table A1, found in the appendix, shows the *a*, *b*, *c*, and *c’* values for each of the NCC/CCW models. The figures that display significant models in the triangle form shown in Figure 5 are contained in the appendix.

The relationships between need for cognitive closure and overall conservatism are similar for men (*a* = 0.12, *p* < 0.01) and women (*a* = 0.13, *p* < 0.001). Comparable relationships between overall conservatism and climate change worry were also found for men (*b* = −0.59, *p* < 0.001) and women (*b* = −0.51, *p* < 0.001). However, men and women differ greatly when it comes to the direct NCC/CCW and mediated NCC/CCW relationships. Women exhibit partial mediation, with *c’* < *c*, (*c* = −0.1, *p* = 0.00575; *c’* = −0.03). Men, on the other hand, exhibit suppression. For men, *c’* > *c*, with *c’* being significant (*c* = 0.05, *p* = 0.351; *c’* = 0.12, *p* < 0.001). Another interesting difference is that the *c* coefficients for men are positive, while for women, they are negative. This partial mediation vs. suppression difference remained when we broke these gender-based mediation models down further by the dimensions of conservatism: social and economic. The Sobel test was used to determine the significance of the mediation effects. The only effects that were significant were social and overall conservatism’s suppressing effect in men. Table A2 and Figure A2 and Figure A3 in the appendix model these relationships visually.

### 3.2. Need for Cognitive Closure, Conservatism, and Solution Support

**Figure 6:**
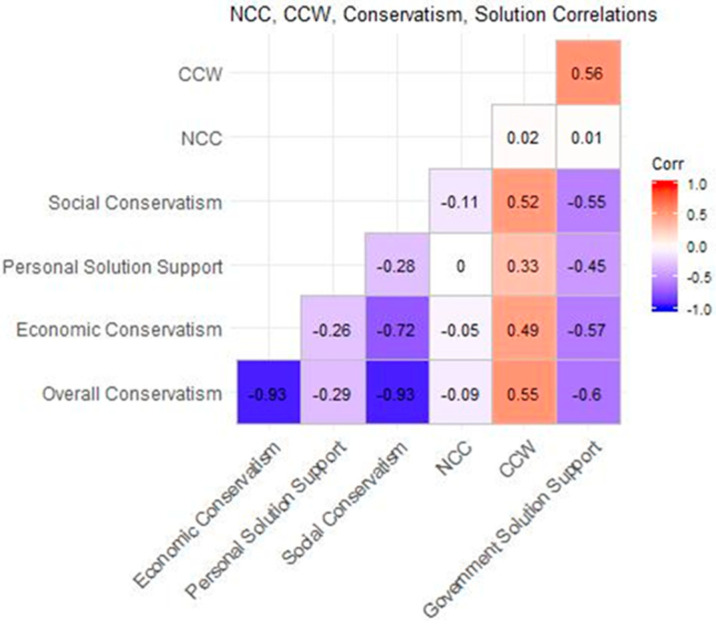
Correlation coefficients (R^2^) for CCW, NCC, different dimensions of conservatism, and level of support for government-based and personal climate solutions. Each of the correlations displayed are significant (*p* < 0.05).

Figure 6 shows correlations between climate change worry, need for cognitive closure, conservatism (overall, social, and economic), and support for both government-based and personal climate solutions. One thing to note from these correlations is that the coefficient for climate change worry and government-based solutions is close to twice that of climate change worry and personal actions (0.56, 0.33). Correlations between need for cognitive closure and these variables were small (i.e., −0.09, 0.02), while correlations between conservatism and solution support were larger (i.e., −0.26, −0.55). To further investigate these relationships, we created models representing the relationships between need for cognitive closure and solution support mediated by the dimensions of conservatism. Table A3 in the appendix shows the coefficients of each of these models.

**Figure A1:**
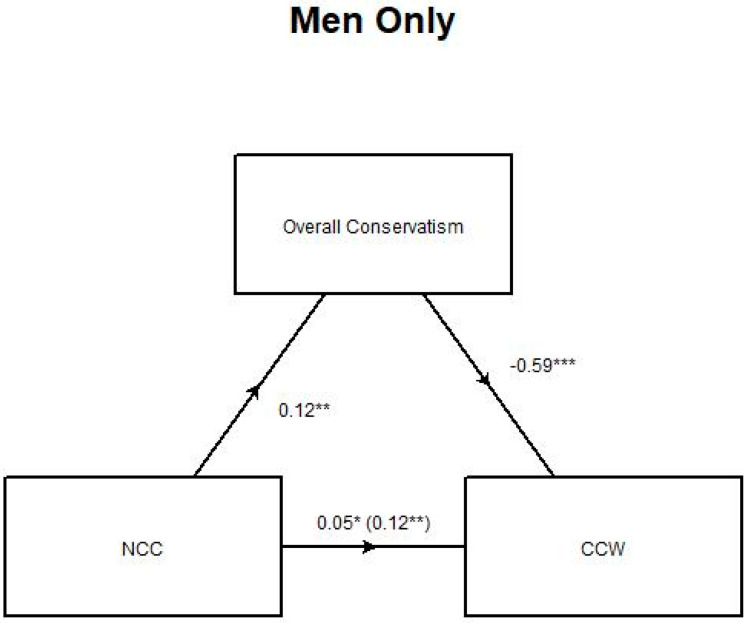
Model of the relationship between NCC and CCW as mediated by conservatism for men. The numbers in this model represent the coefficients of each relationship in a regression model. The *c*’ coefficient is in parentheses. (*** = *p* < 0.01, ** = *p* < 0.05, * = *p* < 0.1).

**Figure A2:**
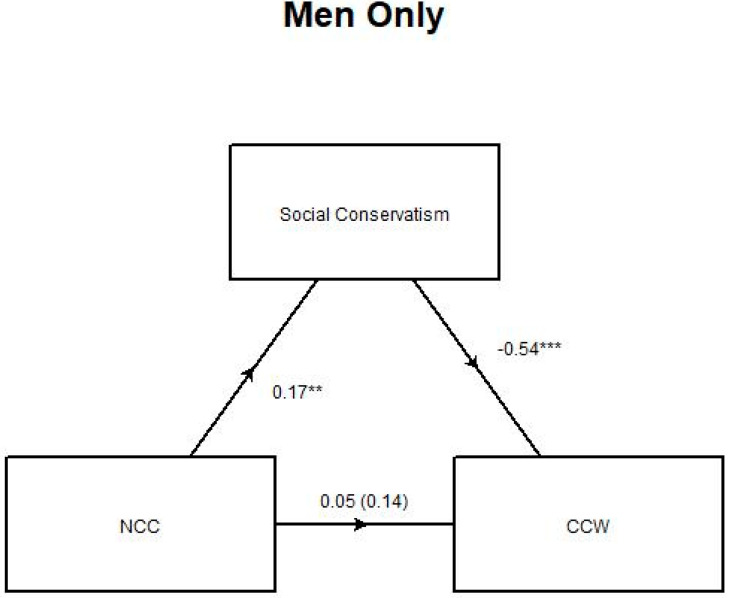
Model of the relationship between NCC and CCW as mediated by social conservatism for men. The numbers in this model represent the coefficients of each relationship in a regression model. The asterisks denote the level of statistical significance of the observed statistics. The convention is *** *p* < 0.001 and ** *p* < 0.05.

**Figure A3:**
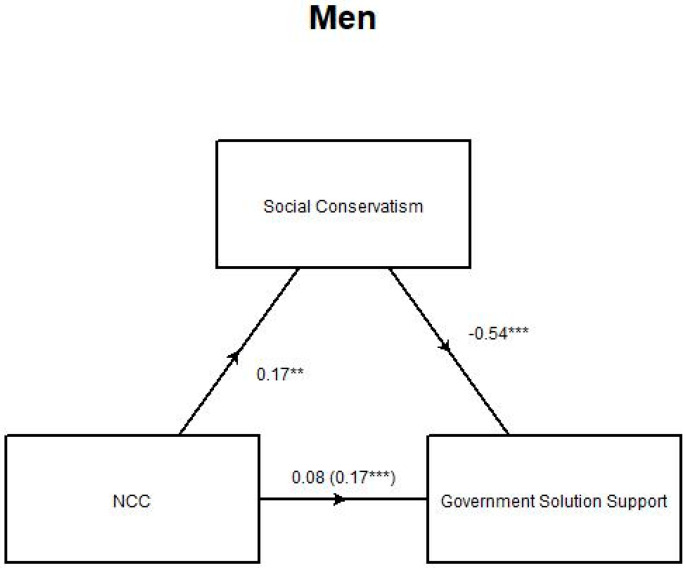
Relationship between NCC and government solution support as mediated by social conservatism for men. The numbers in this model represent the coefficients of each relationship in a regression model. The asterisks denote the level of statistical significance of the observed statistics. The convention is *** *p* < 0.001 and ** *p* < 0.05.

**Figure A4:**
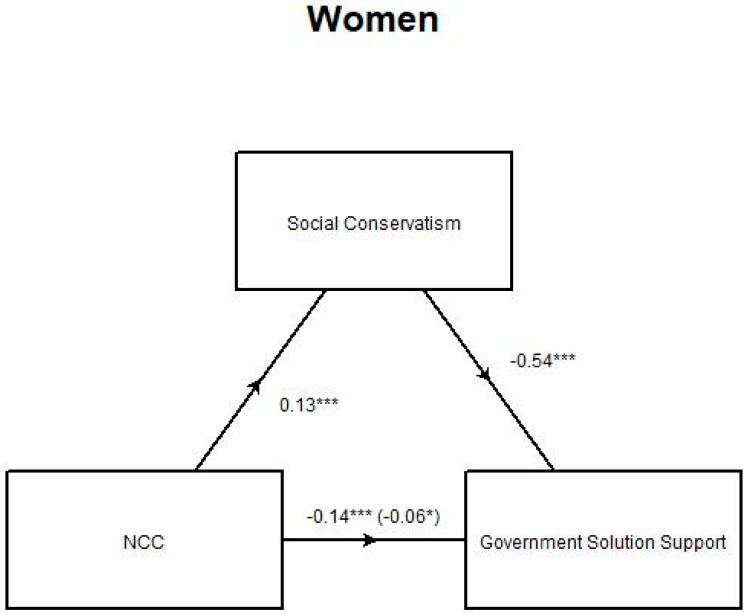
Relationship between NCC and government solution support as mediated by social conservatism for women. The numbers in this model represent the coefficients of each relationship in a regression model. The asterisks denote the level of statistical significance of the observed statistics. The convention is *** *p* < 0.001 and * *p* < 0.01.

**Figure A5:**
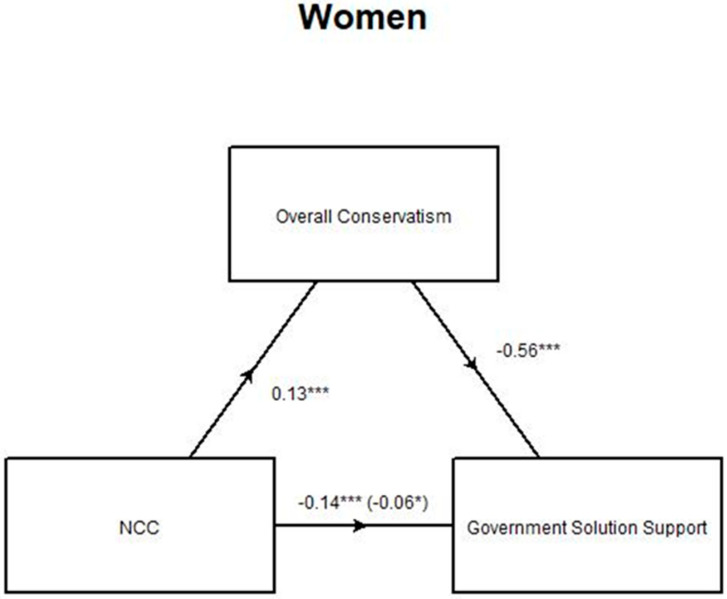
Relationship between NCC and government solution support as mediated by overall conservatism for women. The numbers in this model represent the coefficients of each relationship in a regression model. The asterisks denote the level of statistical significance of the observed statistics. The convention is *** *p* < 0.001 and * *p* < 0.01.

**Figure A6:**
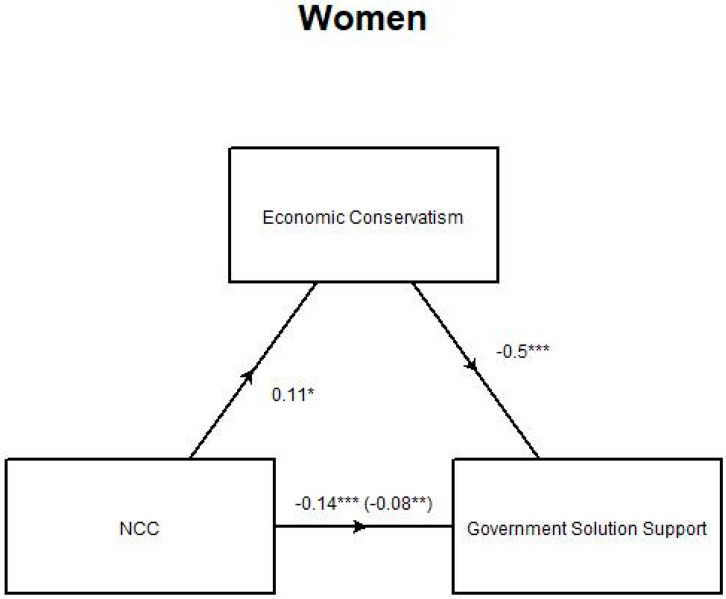
Relationship between NCC and government solution support as mediated by economic conservatism for women. The numbers in this model represent the coefficients of each relationship in a regression model. The c’ coefficient is in parentheses. The asterisks denote the level of statistical significance of the observed statistics. The convention is *** *p* < 0.001, ** *p* < 0.05 and * *p* < 0.10.

Both men and women show an interesting difference in *b* coefficients between solutions. The *b* coefficients for government solutions are larger than for personal actions. For example, in the social conservatism mediator/government solution model, the women’s and men’s coefficients are both −0.54. In the social conservatism mediator/personal solution model, the men’s *b* coefficient is −0.25, and the women’s is −0.27. The *a* coefficients are also fairly similar across genders—NCC and social conservatism is 0.13 for women and 0.17 for men, for example. However, these models exhibit the same partial mediation vs. suppression difference between genders as the previous NCC/CCW models. All types of conservatism function as suppressors in the men’s models. For women, all types of conservatism are partial mediators. Only the effects on government solution support are significant, as shown by Table A4 in the appendix. Table A4 shows that only government-based solutions exhibit a significant conservatism-mediated/suppressed relationship with need for cognitive closure. These significant models are included with the figures in Appendix A (Figure A1, Figure A2, Figure A3, Figure A4, Figure A5 and Figure A6; Table A1,Table A2, Table A3,Table A4, Table A5 and Table A6).

## 4. Discussion

The results of this study show that for college students, overall, men consistently exhibit suppression by conservatism in the relationship between need for cognitive closure and both climate change worry and solution support. This means that an already-existing relationship between NCC and CCW/solution support is strengthened by the presence of conservatism. Women, on the other hand, consistently exhibit partial mediation by conservatism for the NCC/government solution support relationships, but none of the others. Partial mediation means that the presence of conservatism is necessary for there to be any relationship between NCC and government solution support. This gender-based difference has some implications for climate messaging.

For the NCC/CCW relationships, the only significant results were suppression in men by social and overall conservatism. This means that, for men, the presence of conservatism—social, especially—increases the positive effect of need for cognitive closure on climate change worry. Men who are socially conservative and high in NCC are therefore more likely to exhibit high climate change worry than their counterparts who are less socially conservative. Therefore, there is likely a certain group of socially conservative men for whom messages combining uncertainty resolution and the values of social conservativism would be particularly effective. However, message testing research should investigate this further.

Of the two types of solutions presented in this survey study—personal actions and government actions—only the relationships between NCC and government solutions mediated by all dimensions of conservatism were significant. Personal solution support was not significantly related to NCC through conservatism. Within the NCC/government solution relationships, however, the aforementioned gender difference was observed. For men, who exhibited suppression, the presence of conservatism intensified the positive relationship between NCC and government solution support. That is, support for government solutions increases with higher NCC in men, and this increase is stronger when conservatism is present. For women, who exhibited partial mediation, the presence of conservatism plays a vital role in the negative relationship between NCC and government solution support. That is, support for government solutions decreases with higher NCC in women, and conservatism is a necessary link between these two—without it, the relationship is not significant.

## 5. Conclusions

Taken together, these results show that high need for cognitive closure does play a role in climate change worry and solution support, mediated by various dimensions of conservatism. However, this role varies between men and women. While the particular power of social conservatism over economic is consistent with the findings of Panno et al. [10], this study found a significant difference between the genders that was not present in the Panno et al. [10] study. This difference may be present due to the highly politicized and divisive nature of climate change in American society. While environmental issues in general, such as those in the Panno et al. study, are also a topic of debate, climate change is a particularly hot button issue. Thus, it is possible that the use of climate change in the present study resulted in further division than observed in Panno et al. [10]. The present study’s findings can be used to inform audience segmentation and message creation. For example, messages could focus on losses that are results of climate change. For college students, these might include loss of or change to coastlines, spring break destinations, or towns where they aspire to live someday. Further, this message could be expanded to include the uncertainty element and utilize need for cognitive closure, especially for those who have the predictive power of need for cognitive closure increased by social conservatism. A message like this might touch on recent costly disasters, and acknowledge that there is limited uncertainty as to how these disasters will change with a changing climate—but we can take actions to reduce that uncertainty by reducing CO_2_ emissions. The suppression effect seen in men with social conservatism and government solution support means that for some people, combining government action examples with uncertainty resolution may increase support for those government solutions. In that case, the message could emphasize that the level of uncertainty regarding changing disasters can be reduced by taking actions such as a carbon tax or replacing fossil fuels with renewable energy sources—decreasing the likelihood that we could see worsened disasters. However, the mediation effect and negative relationship seen with all dimensions of conservatism in women with regards to government solutions means that this strategy may be more likely to backfire than to work with certain groups of people. Potential audiences for these messages should be studied and targeted carefully. As mentioned previously with the research questions, these messages are meant to resolve uncertainty and increase self-efficacy in those with high need for cognitive closure. Individuals who are low in need for cognitive closure will not be affected by messages about uncertainty, and thus, they are not the focus of the example messages or this study.

It should be noted that these findings are not necessarily applicable to a general population, for two reasons. First, this study used college students as subjects; and while the respondents were somewhat diverse in race, (70% white, 6.5% Latinx, 6.6% black/African-American, and 11.3% Asian/Pacific Islander, see Appendix A for other statistics), this cannot be considered a representative sample. Second, the difference between genders raises some interesting questions. While general worldview does not necessarily strictly follow the traditional gender binary, it is clear from the results that suppression applies to men while partial mediation applies to women. What is unclear is the reason behind this difference.

There are a few avenues for further research based on this study. Of course, one is to investigate the suppression vs. partial mediation difference between the genders. Another is to apply this framework to a more representative sample, to investigate whether these findings are valid beyond a college student sample. This framework would likely be effective in countries aside from the US that have a simple two-party political belief system. Adjustments may need to be made for use in countries such as Italy, where political parties are numerous. Another is to rearrange the mediation models in the solution-based part of the study to learn more about the potential effects of climate change worry on solution support, as a mediator or as an independent variable. Other individual personality differences could be added to these models to determine what other variables may result in the difference in conservative’s function between men and women. In addition, the role of conservatism itself in these relationships could be studied in more detail, to investigate these differences between men and women. Future messaging studies could test each of the aforementioned potential message formats on various populations based on personality and political leaning. Another interesting avenue for further research is the development of a “Need for Climate Change Closure” scale. Such a scale could adapt the pre-existing questions in either the 41-item or 15-item scale so that they ask about climate change specifically. Similarly, while the brevity of the self-reported conservatism [17] scale helps to keep the number of survey questions down and reduces participant fatigue, there are limitations to self-reporting political affiliation. The use of the terms “liberal” and “conservatism” encourage respondents to respond according to the group with which they identify. For example, a respondent may answer “very conservative” because they see themselves as a member of the conservative group of society, where their actual views may fall more centrally on this spectrum. “Political identity” may be a better term for the outcome of the questions in this scale, and this could be an interesting metric for future studies to consider. Additionally, this study did not investigate behaviors such as implementation of individual solutions into one’s lifestyle, or voting behaviors. Future work should build on the present study by looking in to how government solution support relates to voting behaviors. A previous study by Sinatra et al. investigating the outcomes of persuasive messages on college students’ intents to act on climate change showed positive changes in attitudes about climate change and willingness to act, and may serve as a helpful resource in this future avenue of study [19]. The college student age group represents the world’s leaders and voters of tomorrow, making them an important population to consider and study with regards to their support and implementation of vital climate change mitigating actions.

## Supplementary Materials

The following are available online at https://www.mdpi.com/1660-4601/17/15/5619/s1.

## Appendix A

**Table A1:** *a*, *b*, *c*, and *c’* values for models representing the NCC/CCW relationship with overall, social, and economic conservatism as mediators. The asterisks denote the level of statistical significance of the observed statistics.

Gender	Conservatism	*a*	*b*	*c*	*c’*
Both	Overall	0.09 *	−0.55 ***	−0.02	0.03
Men	Overall	0.12 **	−0.59 ***	0.05 *	0.12 **
Women	Overall	0.13 ***	−0.51 ***	−0.1 **	−0.03
Men	Social	0.17 **	−0.54 ***	0.05	0.14
Women	Social	0.13 ***	−0.51 ***	−0.1 **	−0.03
Men	Economic	0.06	−0.54 ***	0.05	0.08
Women	Economic	0.11 *	−0.44 ***	−0.1 **	−0.05

The convention is *** *p* < 0.001, ** *p* < 0.05, and * *p* < 0.10.

**Table A2:** Indirect effect (a*b) values and Sobel Test t and *p* values.

Gender	Conservatism	Sobel *t* Value	*p*-Value
Both	Overall	1.26	0.21
Men	Overall	2.74 **	0.006
Women	Overall	1.05	0.21
Men	Social	3.05 **	0.002
Women	Social	1.05	0.29
Men	Economic	1.77	0.07
Women	Economic	1.55	0.11

The convention is ** *p* < 0.05, and * *p* < 0.10.

**Table A3:** Mediation model coefficients for the relationships between NCC and government/personal solution support, with dimensions of conservatism as mediator/suppressor. The asterisks denote the level of statistical significance of the observed statistics.

Gender	Conservatism	Solution Type	*a*	*b*	*c*	*c’*
Men	Overall	Personal	0.12 **	−0.24 ***	−0.01	0.02
Men	Overall	Government	0.12 **	−0.61 ***	0.08	0.15 **
Men	Social	Personal	0.17 **	−0.25 ***	−0.01	0.04
Men	Economic	Personal	0.06	−0.2 ***	−0.01	0.01
Men	Social	Government	0.17 **	−0.54 ***	0.08	0.17 ***
Men	Economic	Government	0.06	−0.59 ***	0.08	0.11 *
Women	Overall	Personal	0.13 ***	−0.29 ***	−0.05	−0.01
Women	Overall	Government	0.13 ***	−0.56 ***	−0.14 ***	−0.06 *
Women	Economic	Personal	0.11 *	−0.26 ***	−0.05	−0.02
Women	Economic	Government	0.11 *	−0.5 ***	−0.14 ***	−0.08 **
Women	Social	Personal	0.13 ***	−0.27 ***	−0.05	−0.01
Women	Social	Government	0.13 ***	−0.54 ***	−0.14 ***	−0.06 *

The convention is *** *p* < 0.001, ** *p* < 0.05, and * *p* < 0.10.

**Table A4:** Sobel *t* and *p* values for solution support. The asterisks denote the level of statistical significance of the observed statistics.

Gender	Conservatism	Solution Type	Sobel *t* Value	*p*-Value
Men	Overall	Personal	0.47	0.64
Men	Overall	Government	3.46 ***	0.0005
Men	Social	Personal	0.71	0.48
Men	Economic	Personal	0.11	0.912
Men	Social	Government	3.56 ***	0.0003
Men	Economic	Government	2.49 **	0.012
Women	Overall	Personal	0.33	0.74
Women	Overall	Government	1.97 *	0.05
Women	Economic	Personal	0.59	0.56
Women	Economic	Government	2.42 **	0.015
Women	Social	Personal	0.39	0.69
Women	Social	Government	2.01 *	0.044

The convention is *** *p* < 0.001, ** *p* < 0.05, and * *p* < 0.10.

**Table A5:** Ethnicity Counts.

Ethnicity	Count
White	772 (70%)
Latinx	73 (6.5%)
Black/African American	74 (6.6%)
Asian/Pacific Islander	126 (11.3%)
Other	41
Prefer Not to Answer	25

**Table A6:** Religion Counts.

Religion	Count
Jewish	14
Christian-Catholic	160
Christian-Protestant	395
Buddhist	17
Hindu	29
Muslim	21
Agnostic/Atheist	277
Other	82
Prefer Not to Answer	115

Note: many of the “other” responses listed specific Protestant denominations, i.e., Presbyterian, Baptist, Methodist, etc.
