# Supplementary material for: Investigating Connections between Need for Cognitive Closure and Climate Change Concern in College Students
Source: Int J Environ Res Public Health. 2020 Aug 4;17(15):5619. doi: 10.3390/ijerph17155619 (PMC7432500; doi:10.3390/ijerph17155619)
Supplement: Supplementary file 1 [file ijerph-17-05619-s001.pdf]

International Journal of
Environmental Research and Public Health

MDPI

Mediation Modeling

Mediation analysis was heavily used in this study. Mediation is a form of regression modeling where a third variable plays a key role—a link in the chain, so to speak—between the independent and dependent variables. There are four path, or regression, coefficients in a mediation model:

a, which displays the relationship between the independent variable and the mediating variable;

b, which displays the relationship between the mediating variable and the dependent variable;

c, which is the direct relationship between the independent variable and the dependent variable;

c′, which represents the relationship between the independent and dependent variables when the mediating variable, or mediator, is introduced to the model.

The product of a × b represents the effect of the mediator. When working with three variables, mediation models are usually displayed in a triangle format, with the mediating variable serving as the tip of the triangle, and the path coefficients on the lines connecting the variables. In mediation modeling, we are looking for the difference between c and c′ where $c' = c - (a \times b)$. If $c = 0$, this indicates complete mediation, where the mediator takes complete responsibility for the relationship between the independent and dependent variables. If $c' < c$, partial mediation is occurring, which means that the mediator plays a role in the independent/dependent variable relationship, but the direct effect maintains some of its role. Many, perhaps most, mediation relationships exhibit partial mediation. Finally, if $c' = c$, the proposed mediator actually has no effect on the relationship and there is no mediation. Coefficients are considered significant at $\alpha = 0.05$. The 95% confidence interval of a × b is used to assess whether the mediation value could have occurred due to random chance. The product of a × b is equal to the effect of the mediator within the model. If zero is not in the 95% confidence interval for a × b, we can conclude that the indirect effect, or the amount of mediation, is different from zero.

While $c' < c$ means that partial mediation is occurring, it is also possible that $c' > c$ could be observed. This indicates suppression, which means that the presence of the mediating variable increases the magnitude of the direct relationship. That is, the suppressor intensifies the predictive power of the independent variable with regards to the dependent variable. Suppression is also indicated when the directionalities of c and c′ are different (i.e., one is negative while the other is positive).
